# „Wider den nächtlichen Unfug“ – Die Bielefelder Straßenbeleuchtung von 1853 bis in die 1880er Jahre als versicherheitlichende Infrastruktur und Mittel der Selbstdisziplinierung

**DOI:** 10.1007/s00048-024-00386-1

**Published:** 2024-05-24

**Authors:** Paul Franke

**Affiliations:** Institut für Sozial- und Wirtschaftsgeschichte, Philipps-Universität Marburg, 35032 Wilhelm-Röpke-Straße 6, Marburg, Deutschland

**Keywords:** Stadtgeschichte, Historische Sicherheitsforschung, Raumgestaltung, Gouvernementalität, Securitazation, Urban history, Historical security studies, Urban design, Securitazation, Gouvernementalité

## Abstract

Dieser Artikel beschäftigt sich mit dem Wandel von Sicherheitsbedürfnissen und technologischen Möglichkeiten im Zuge der Industrialisierung anhand der Etablierung der Bielefelder Straßenbeleuchtung im 19. Jahrhundert. Er zeigt, wie die Entwicklung von der Öl- hin zur Gaslaterne zusammenfiel mit einem veränderten Sicherheitsbedürfnis im städtischen Bürgertum. Mit den technischen Möglichkeiten der Gasbeleuchtung konnte nunmehr der nächtliche städtische Raum durchdrungen werden und dadurch einerseits marginalisierte und als Sicherheitsrisiko wahrgenommene Personengruppen sichtbar gemacht sowie auf der anderen Seite dem Licht inhärente disziplinierende Bestrebungen bei den Bürger*innen internalisiert werden. Waren die städtischen Bevölkerungen der Vormoderne und des frühen 19. Jahrhunderts Laternen noch skeptisch oder ablehnend gegenübergestanden, so war bereits Mitte des 19. Jahrhunderts ihre Installation Teil von dezidiert stadtbürgerlichen Forderungen nach mehr persönlicher, ökonomischer und Verkehrssicherheit. So wandelte sich die Laterne von einem Instrument des vormodernen Sichtbarmachens zu einem Instrument beständiger Sichtbarkeit in der Moderne. Dies führte zu neuen Konfliktlinien, da die Expansion der Beleuchtung nicht so umfassend verlief wie vom Stadtbürgertum gefordert. Hier zeigt das Beispiel Bielefeld neben Wandel und Konjunkturen von Versicherheitlichungsbedürfnissen im Zuge der Industrialisierung auch, dass eine Internalisierung obrigkeitsstaatlicher Vorstellungen keinesfalls die Abwesenheit von Konflikt bedeutete. Anhand von Verwaltungsakten und Eingaben wird aus der Perspektive der Historischen Sicherheitsforschung unter Rückgriff auf Foucaults Konzept der Gouvernementalität die Geschichte der Bielefelder Straßenbeleuchtung in den Kontext eines größeren Wandels von Sicherheit, Technik und urbaner Raumgestaltung gesetzt. Die Ergebnisse zeigen, dass Technik- und Infrastrukturgeschichte wesentlich dabei helfen, die Erkenntnisse der Historischen Sicherheitsforschung zu vertiefen und zu kontextualisieren. Techniknutzung und -erwartungen waren durch eine bisweilen prekäre Allianz verschiedener Akteursgruppen ein wesentlicher Bestandteil eines neuen Sicherheitsverständnisses als auch der sozialsegmentierten Ordnung des urbanen Raums.

## Abstract (en)

This article deals with the change in safety requirements and technological possibilities in the course of industrialization by looking at the establishment of street lighting in Bielefeld in the 19th century. As will be shown, the development from oil to gas lanterns coincided with a change in the security needs of the urban middle class. It was the technical possibilities of gas lighting to penetrate the urban space at night that made marginalized groups of people who were perceived as a security risk visible. This, together with the bourgeois internalization of the disciplinary effects of light, made this infrastructure possible in Bielefeld. While the urban populations of the pre-modern and early 19th century were still skeptical or dismissive of lanterns, by the mid-19th century their installation was already part of decidedly urban bourgeois demands for more safety in the areas of personal, economic and traffic safety. The lantern thus changed from an instrument of pre-modern visibility to an instrument of constant visibility in the modern age, which at the same time led to new lines of conflict when the expansion and extension of lighting was not as comprehensive as demanded by the urban bourgeoisie. In addition to the changes and conjunctures of security needs in the course of industrialization, Bielefeld also shows that an internalization of the concept of sovereignty by no means meant the absence of conflict. On the basis of administrative acts and petitions, the history of Bielefeld’s street lighting is placed in a larger transformation of security, technology and urban spatial design from the perspective of historical security research, drawing on Foucoult’s concept of gouvernmentalité. The results show that the history of technology and infrastructure can significantly deepen and contextualize the findings of historical security research. The use and expectations of technology were an essential part of a new understanding of security, as well as the socially segmented organization of urban space through a sometimes precarious alliance of different groups of actors.

## Technikgeschichte und Historische Sicherheitsforschung im Kontext der Lokal- und Stadtgeschichte

Das Beherrschen der nächtlichen Dunkelheit ist ein historischer Prozess, der technologische Möglichkeiten, politische Ansprüche, soziale und sozio-ökologische Konflikte mit sich brachte. Die Überwindung, Domestizierung und Kommerzialisierung der Nacht war in den letzten zwei Jahrhunderten gleichzeitig ein zentrales Element der Sicherheitskultur und sichtbares, leuchtendes Zeichen von Modernität und Zivilisation. Das Überwinden der Beeinträchtigung des Sehsinns und des scheinbar natürlich gegebenen zirkadianen Rhythmus durch Technologie schien ein deutliches Anzeichen für die Möglichkeiten, die Umwelt beherrschbar zu machen (Jütte 2000: 65–83; Bronfen 2008: 37–139; McMahon 2018: 119–121). Doch eine bloße Erfolgsgeschichte ist die Durchdringung und Beleuchtung der Nacht nicht: Seit den 2000ern wird der Verlust nächtlicher Dunkelheit durch „Lichtverschmutzung“ aus sozialer, infrastruktureller, aber nicht zuletzt ökologischer Perspektive problematisiert (Hölker et al. 2010).

Der Biopsychologe Peter Walschburger argumentierte 2022 in einem Interview, dass die Dämmung oder Abschaltung der umfassenden Beleuchtung in Städten „positive Effekte“ hätte – ökonomische, sicherheitspolitische, aber auch psychologische. Die Energiekrise wäre eine Chance, „unsere Hygiene des Tag-Nacht-Lebens zu verbessern“. Er sprach sich für weniger Licht und Technologie und stattdessen für verstärkten menschlichen Einsatz aus, um das Sicherheitsgefühl zu stärken – etwa in Form der Wiedereinführung von „Schupos“; also von Polizeibeamt*innen, die in der Stadt patrouillierten und so als Schnittstelle zur Bevölkerung agierten.^1^

Kulturkonservative Kritik, ästhetische Problematisierung und ökologische Bedenken werden dabei aber nicht nur im 21. und 20. Jahrhundert, sondern bereits im 19. Jahrhundert greifbar (Milan 1998: 122). Ute Hasenöhrl hat in ihrer Arbeit aufgezeigt, dass die Expansion der Beleuchtung und der Umgang mit Dunkelheit mit vielschichtigen politischen und sozialen Konflikten verbunden sind, die es zu untersuchen gilt (Hasenöhrl 2014: 100–125).

Darin liegt die Chance für eine Untersuchung der Straßenbeleuchtung als Technologie und Infrastruktur im Rahmen der stärker diskutierten Historischen Sicherheitsforschung (Rieker & Zimmermann 1997: 46–85; Conze 2023: 27–32). Ausgangspunkt dieses Artikels ist daher die historische Betrachtung der Straßenbeleuchtung als Teil einer größeren Entwicklung um Sicherheitsdiskurse in der Moderne. Anhand der Bielefelder Straßenbeleuchtung wird auf einer lokalhistorischen Ebene exemplifiziert, wie die Straßenbeleuchtung von einem obrigkeitsstaatlichen Projekt zu einem vom Stadtbürgertum mitgetragenen Sicherheitsdispositiv wurde. Als durch Industrialisierung, Urbanisierung, administrative Verschiebungen und technologischen Wandel neue Sicherheitsbedürfnisse im Bürgertum der Bielefelder Alt- und Neustadt aufkamen, wurde die Straßenbeleuchtung als Mittel angesehen, darauf zu reagieren. Zwischen den 1850er und 1890er Jahren internalisierte das Stadtbürgertum die disziplinierenden Aspekte der Straßenbeleuchtung, was wiederum zu neuen Konflikten mit dem Magistrat als politischer Institution führte. In Bielefeld zeigen sich daher auf einer Mikroebene historische Brüche und Kontinuitäten in der Beleuchtungsgeschichte sowie die Möglichkeit, sie im Rahmen der Versicherheitlichungsgeschichte – abseits einer postulierten linearen Entwicklung – zu untersuchen.

## Sicherheitsgeschichte und Historische Sicherheitsforschung

Die Historische Sicherheitsforschung hat in den letzten Jahrzehnten eine deutliche Ausweitung erfahren (Conze 2005: 357–380; Conze 2023: 30–32; Lübbe 1989: 5–29; Göllnitz & Mecking 2023: 3). Nachdem sie den Fokus anfänglich auf Diplomatie und Staat legte, nahm sie spätestens seit dem *cultural turn* auch andere Zusammenhänge in den Blick. Darunter etwa die Bedeutung subjektiver Wahrnehmungsprozesse von „sicher“ und „Sicherheit“, die Auseinandersetzung mit diesen Begriffen selbst sowie die Unterschiede darin, welche Sicherheitsbedürfnisse in Gesellschaften, sozialen Gruppen und anderen Akteurszusammenhängen existierten und artikuliert wurden (Weinhauer 2019: 368–370; Conze 2018: 21–46). Dabei nahm der Begriff der „Versicherheitlichung“ eine wichtige Rolle ein. Ursprünglich ein Konzept der internationalen Beziehungen und in den 1990er Jahren geprägt (*Securitazation*), nutzt die Geschichtswissenschaft diesen Begriff, um die Herstellung von Sicherheit als Prozess zu analysieren. Sicherheitsgeschichte und Historische Sicherheitsforschung ist daher weniger bemüht, Sicherheit historisch zu quantifizieren, sondern Sicherheit und Unsicherheit als Begriffe, Emotionen, politische Themen und nicht zuletzt als praktische Projekte zu historisieren.^2^

Eckart Conze hebt dabei heraus, dass es spätestens seit der Frühen Neuzeit eine Tradition gibt, als „unbeherrscht“ wahrgenommene Räume zu versicherheitlichen (Conze 2018: 82–100). Martin Göllnitz und Sabine Mecking haben in diesem Zusammenhang darauf verwiesen, dass Versicherheitlichung als Prozess über ein sich historisch wandelndes Repertoire an Instrumenten verfügt, die Gesellschaften als möglich erscheinen und ihnen zur Verfügung stehen (Göllnitz & Mecking 2023: 16–21).

Diese Perspektive auf (Un‑)Sicherheit macht die Historische Sicherheitsforschung anschlussfähig an die Infrastruktur- und Technikgeschichte, welche die symbolisch-semantische Aufladung und soziokulturelle Praktiken mit und um Technologie in ihre Untersuchung integriert hat (Barth 2008: 12–14). Wesentlich vorangetrieben wurde diese Perspektive auf Infrastruktur durch den Historiker Dirk van Laak, der in seiner Forschung zur Historisierung von Infrastrukturen aufrief und dafür plädierte, diese als „Steuerinstrumente“ zu begreifen. Infrastruktur sei potenziell fähig, zu vereinnahmen oder gar zu kolonisieren. Er betonte, dass dies nicht zwangsläufig ein zielgerichteter Prozess sei, sondern Infrastruktur eine eigene Dynamik entfalten könne (Van Laak 2001: 369–372). Van Laak sieht hier eine wichtige Schnittstelle von Technik- und Infrastrukturgeschichte, da die Technik moderner Infrastrukturen und ihre Verfügbarkeit für die Massengesellschaft das Potenzial enthalte, die Bedürfnishierarchie der Individuen radikal umzuschichten (ebd.: 372). Er verweist darauf, dass ein durch sie geschütztes oder verfolgtes „Gemeinwohl“ interessengeleitet war und Infrastrukturen damit auch „mit Vereinheitlichung oder sogar entmündigender Disziplinierung“ (ebd.: 373) einhergingen. Infrastrukturen werden somit in der neusten Forschung als Schnittstellen und Produkt soziokultureller, sozio-ökologischer und technologischer Systeme und Prozesse betrachtet (Pritchard 2011: 19–20; Bonan & Occhi 2023: 7–9). Frédéric Graber weist im selben Zusammenhang darauf hin, dass Infrastrukturen und ihre Etablierung keinem strikten Top-down-Mechanismus folgen (Graber 2023: 130). Gleichzeitig betont er, dass gerade staatstragende Infrastrukturen immer auch Restriktionen, Ungleichheiten und Hierarchisierung enthalten. Infrastrukturen sind damit – selbst wenn gemeinnützig – nie tatsächlich für alle (ebd.: 129). Es ist also historisch wichtig, Infrastruktur- und Stadtgeschichte gemeinsam unter dieser Perspektive zu betrachten (Lenger 2009: 79).

## Die Nacht als Terra incognita?

Die Versicherheitlichung und damit Disziplinierung von Akteur*innen und Räumen durch Infrastrukturen ist für die nächtliche Straße beziehungsweise den nächtlichen öffentlichen Raum zentral und seit der Frühen Neuzeit unter sich verändernden politischen, kulturellen und technologischen Vorzeichen ein Projekt verschiedener Obrigkeiten und Gesellschaften. Die Nacht stellte ein komplexes Gefüge aus Zuschreibungen und Zuständen dar; sie war mehr als dunkel. Sie war als bedrohlich und irrational-weiblich kodiert, während der Tag als sicher und rational-männlich gesehen wurde (Bronfen 2008: 9–37; Koslofsky 2011: 57–174). Die Nacht veränderte die Lebenswelt der Zeitgenoss*innen, schuf Unordnung und weckte Ängste vor dem Fremden, das sich in ihrem vermeintlichen Chaos formierte (Baldwin 2012: 1–14). Normative Ordnungen ermahnten daher „ehrliche Bürger*innen“, sie möglichst zu meiden (Bouman 1987: 8; Beaumont 2016: 15–30). Die Nacht schien gerade die Stadt zu transformieren; selbst für Einheimische wurde sie dann eher zur „terra incognita“ (Bouman 1987: 9). Sie war scheinbar herrschaftsfrei und damit chaotisch (Lenger 2014: 251). Schivelbusch verbindet die Nacht daher nicht unzutreffend mit für die politische Macht unbeherrschtem, zu durchdringendem Raum: „Aus der Ordnungsperspektive des Absolutismus betrachtet, erschienen die noch nicht von der Polizei kontrollierten und modernisierten Pariser Straßen wie ein Dschungel.“ (Schivelbusch 1983: 86).

Gleichsam haben historiografische Betrachtungen von Nacht und Beleuchtung dieses einseitige Narrativ der Nacht und der nächtlichen Dunkelheit als Negativfolie zum Tag als Problemraum thematisiert. In seiner Studie zum frühneuzeitlichen London präsentiert Matthew Beaumont die nächtliche Straße als gefährlich, aber auch als Freiraum der Marginalisierten und stellt das Narrativ der Gefährlichkeit sogenannter „Nightwalker“ infrage. Er verweist darauf, dass es eine dezidiert politische Perspektive ist, die nächtliche Straße als Sicherheitsproblem außerhalb regulierter Herrschaft zu begreifen (Beaumont 2016: 5–11).

In seiner empirisch reichen Studie zu Zürich in der Frühen Neuzeit bis in das 19. Jahrhundert hat Christian Casanova bereits gezeigt, dass die Nacht als Rückzugs- und Erlebnisraum denen zur Verfügung stand, die sich gegen etablierte Macht‑, Wirtschafts- oder Geschlechterverhältnisse positionierten oder sich ihnen temporär zu entziehen suchten. Regulierungsambitionen der Obrigkeit waren daher auch gegen bestimmte Gruppen gerichtet, die außerhalb der sichtbaren und damit legitimen Gesellschaft verortet wurden (Casanova 2007: 84).

Auch die Beleuchtung, die seit Wolfgang Schivelbuschs Arbeit als Disziplinierungstechnologie analysiert wird, ist immer wieder anders interpretiert worden. So hat Chris Otter in seinem Buch zu Seh- und Beleuchtungspraktiken im 19. Jahrhundert darauf verwiesen, dass die künstliche Beleuchtung und das neue Sehen des viktorianischen Zeitalters nicht nur die Möglichkeit zur Disziplinierung, sondern auch neue Freiheiten eröffnete (Otter 2008: 1, 100). David Nye hat neben der europäischen auch die US-amerikanische künstliche Beleuchtung untersucht und dabei nicht nur Unterschiede in der Implementierung der Beleuchtung, sondern zudem in ihrer Interpretation festgemacht (Nye 2018: 4). Er betont, dass Beleuchtung den US-amerikanischen Stadtraum hierarchisierte und strukturierte. Gerade das Fehlen von Beleuchtung hätte bestimmte soziale Probleme und Gruppen unsichtbar gemacht (ebd.: 107). Nye hebt den Zusammenhang zwischen Beleuchtung und politischer Macht hervor, wendet sich aber entschieden gegen Modernisierungsautomatismen oder eine zu starke Pfadabhängigkeit.

Dieser Artikel greift die angesprochenen Problematiken und die bisherige Forschung auf. In Bielefeld – einer Stadt, die Mitte des 19. Jahrhunderts einen rasanten Industrialisierungsprozess durchlief – werden viele abseits der Metropolen stattfindenden Prozesse greifbar. Dass sich die Beleuchtungspraktiken von mittleren und kleineren Industriestädten wesentlich von denen der Metropolen unterschieden, hat bereits Bouman argumentiert und dabei auf die Notwendigkeit verwiesen, erstere gesondert zu untersuchen (Bouman 1987: 13–14). Otter und Nye haben mit ihren Arbeiten zudem gezeigt, dass nationale beziehungsweise globale Unterschiede auch andere Beleuchtungs- und Sehpraktiken hervorbrachten. Die Geschichte der Straßenbeleuchtung in Bielefeld ist somit mehr als eine Lokalgeschichte, sondern erweitert die Sicherheits‑, Infrastruktur- und Technikgeschichte um eine wichtige Fallstudie, die Urbanisierung, Industrialisierung, technische, ökonomische wie auch sicherheitshistorische Grundlagen für einen Wandel in der Beleuchtungspraktik zusammenbringt. Die ostwestfälische Industriestadt exemplifiziert dabei, dass das Stadtbürgertum angesichts dieses Wandels die im 18. Jahrhundert vom Obrigkeitsstaat verfolgte Lösung der Straßenbeleuchtung nicht nur als Forderung übernahm, sondern sie sich zu eigen machte und zur Selbstdiziplinierung ebenso wie zur Versicherheitlichung ihrer Lebensräume nutzte.

## Zwingender Blick: Ansatz, Perspektive, und Zugriff

Dieser Artikel möchte Straßenbeleuchtung im Hinblick auf Disziplinierung und Durchsetzung von Herrschaft in Räumen untersuchen, die durch sozioökonomische Prozesse als risikobehaftet imaginiert wurden. Damit verbunden ist die Frage nach der Internalisierung und der Pfadabhängigkeit der modernen Straßenbeleuchtung. Als Beispiel soll die Bielefelder Straßenbeleuchtung dienen, die sich als Gegenstand dafür besonders eignet, da die Infrastrukturgeschichte der ostwestfälischen Stadt in Hinblick auf die Straßenbeleuchtung bereits zu einem guten Teil erschlossen ist, weiterführende Fragestellungen der Technikgeschichte mit ihren kulturellen, sozialen und sicherheitshistorischen Ausrichtungen bisher aber kaum gestellt wurden.

Bielefeld bietet die Möglichkeit, die Geschichte der Straßenbeleuchtung als disziplinierende Sicherheitstechnologie abseits ihrer besonderen politisch-repräsentativen Bedeutung für Metropolen wie Berlin, Paris oder London zu schreiben, die sehr viel mehr in der Tradition der Illumination zu Repräsentationszwecken standen. Anhand von Bielefeld als idealtypischer Stadt der deutschen Industrialisierung im 19. Jahrhundert können daher Muster herausgearbeitet werden, die über die Lokal- und Regionalgeschichte hinausgehen. Mit ähnlichen Argumenten hat der für die Geschichte der Straßenbeleuchtung prägende Historiker Mark J. Bouman Bochum als Untersuchungsgegenstand gewählt – eine Stadt, die in Bezug auf Industrialisierungsvorgänge sowie Ausdehnung und Struktur der Straßenbeleuchtung mit Bielefeld sehr gut vergleichbar ist (Bouman 1987: 14–15). Gleichzeitig zeigt sich in Bielefeld, dass das Schaffen von Infrastruktur, Versicherheitlichung und sozialer Disziplinierung nicht ein einfacher Top-down-Prozess, sondern vielschichtig war.

Um dem Wandel der Straßenbeleuchtung von sichtbar gewordener politischer Durchdringung des städtischen Raumes hin zu internalisierter Selbstdisziplinierung nachzugehen, greift der Artikel den Vorschlag von Conze auf, das Konzept der Gouvernementalität des Philosophen Michel Foucault zu nutzen (Conze 2018: 101–107).

Es muss dabei betont werden, dass das Konzept der Sozialdisziplinierung in der Forschung zur Frühen Neuzeit sehr gut etabliert ist und auf den spürbar wachsenden Anspruch der Obrigkeit zwischen dem 16. und dem 19. Jahrhundert verweist, möglichst viele Lebensbereiche der Untertan*innen und der Bevölkerung zu reglementieren und zu disziplinieren. Maßgebend hierfür war Gerhard Oestreich, der einen Wandel der frühneuzeitlichen Sozialregulierung (über normative Vorschriften) zur Sozialdiszipinierung (Steuerung des öffentlichen Lebens) des Absolutismus nachweist (Oestreich 1969: 179–197; Casanova 2007: 23).

Der Historiker Christian Casanova verweist seinerseits darauf, dass Foucaults Ansätze zur Disziplinierung zwar historisch-empirisch kaum haltbar sind. Er betont aber auch die Möglichkeit der Erweiterung und Anpassung der Theorie – vor allem bezüglich der materiellen Dimension durch Objekte im Raum, die Hinterfragung des Bottom-up-Paradigmas einer einseitigen Durchsetzung der Obrigkeit sowie der Rolle des Scheiterns und der Modifikation (Casanova 2007: 28–29).

Gerade die Rolle des Scheiterns sowie die Gefahr der Einseitigkeit bei der Etablierung von sozialen Disziplinierungsmaßnahmen wurde in Bezug auf die nächtliche Dunkelheit nicht nur von Casanova, sondern auch jüngst von Margo De Koster und Herbert Reinke betont (De Koster & Reinke 2023: 46).

Dieser Aufsatz möchte ausgehend von dieser Kritik Foucaults Grundannahmen für die Analyse nutzen, sie aber entsprechend methodisch anpassen. Eine wichtige Grundannahme Foucaults, dass Disziplinierung durch die Staatsmacht mit dem Sichtbarmachen zu tun hat, steht dabei besonders im Vordergrund. „Die Durchsetzung der Disziplin erfordert die Einrichtung des zwingenden Blicks: eine Anlage, in der die Techniken des Sehens Machteffekte herbeiführen und in der umgekehrt die Zwangsmittel die Gezwungenen deutlich sichtbar machen“ (Foucault 1994: 221). Als solche inkludiert Foucault explizit Technologien und Geräte der Beleuchtung (ebd.: 221).

In diesem Sinne machte die Straßenlaterne erkennbar, was vorher nur sehr eingeschränkt wahrnehmbar war und regulierte damit sowohl den Raum als auch Menschen in ihrer Zeit- und Raumwahrnehmung. Ein zweiter wesentlicher Punkt ist, dass Straßenbeleuchtung und Laterne zur Selbstregulierung und zum Vorantreiben der Disziplinierung dienten. Das Wissen, sichtbar zu sein, war nach Foucault Zwangsmaßnahme und Mittel zur Verhaltensmodifikation, denn wer der Sichtbarkeit unterworfen ist und darum weiß, übernimmt die Zwangsmittel der Macht und spielt sie gegen sich selbst aus. Er internalisiert das Machtverhältnis, in welchem er beide Rollen gleichzeitig spielt, und wird so zum Prinzip seiner eigenen Unterwerfung (Foucault 1992: 260).

Die Bedeutung der Laterne als Sicherheitsdispositiv rührt auch daher, dass sie oberflächlich zwar das Individuum, letztlich aber die Gesellschaft zum Ziel hat. Sicherheitsdispositive reglementieren dabei nicht Einzelne, sondern zielen laut Foucault „auf die Sicherheit des Ganzen vor seinen inneren Gefahren“ (Michel 2005: 12). Ein Problem wie die nächtliche Dunkelheit ist demnach nicht individuell zu lösen, sondern eine gesellschaftliche Aufgabe (ebd.: 11–13). Dies führte dazu, dass die Bielefelder Stadtbürgerschaft eben nicht nur die als ortsfremd und damit gefährlich imaginierten Arbeiter*innen zu regulieren suchte, sondern auch sich selbst regulierte, indem sie ihre Handlungen und Sicherheitsvorstellungen anpasste.

Dabei sind Gouvernementalität und Selbstdiziplinierung in Bezug auf Sicherheitsdynamiken verbunden. Gouvernementalität war nicht nur darauf ausgerichtet, Individuen durch ständige panoptische Kontrolle zu disziplinieren, sondern Räume und Praktiken zu konstruieren, die die Versicherheitlichung durch Verhaltensanpassung im Sinne der Befolgung eigener Interessen nahelegte (Purtschert et al. 2008: 7–8, 12–14; Frohmann 2017: 285–287). Gouvernementale Politik sorgte so für eine scheinbar natürliche Anpassung von Selbstinteressen und Vorstellungen von persönlicher körperlicher, ökonomischer, sozialer und hygienischer Sicherheit der Bürger*innen an scheinbar gesellschaftliche Notwendigkeiten (der Beherrschung der nächtlichen Straße) (Finzsch 2002: 258–261).

Als Quellen werden, an diese Überlegungen anschließend, städtische Verwaltungsakten genutzt, die neben Planungen und Anweisungen zu einem nicht geringen Teil auch Eingaben von Bürger*innen enthalten. An diesen zeigt sich, dass die Bürger*innen an tradierte Ordnungsmuster anknüpften und es zu einer Annäherung der Positionen von Obrigkeit und Bevölkerung kam. Laut Casanova war dies für den Erfolg der Sozialdisziplinierung auch in Städten, etwa Zürich, entscheidend (Casanova 2007: 34–35, 459). Gleichzeitig offenbaren sich hier neue Konflikte, als Stadtbürger*innen ihren Wunsch nach Gaslicht und Regulierung des Raums in fordernder Weise stellten. Es ergibt sich ein differenziertes Bild von Aushandlungsprozessen in der sich industrialisierenden Stadt des 19. Jahrhunderts, in der Konflikte entstanden, weil Anwohner*innen gouvernementale Politik soweit internalisierten, dass die stadtplanerischen und ökonomischen Möglichkeiten der Politik diese nicht gänzlich zu befriedigen vermochten. Das hing mit einem Wandel der Beleuchtungspraktiken, aber auch der Sicherheitswahrnehmungen zusammen.

Der Untersuchungszeitraum umfasst die Periode der beginnenden 1850er Jahre bis in die 1880er Jahre. In Bielefeld war der rund 30 Jahre umfassende Zeitraum geprägt von der Etablierung und Erweiterung der Gaswerke sowie der konstanten Erweiterung der Straßenbeleuchtung. So fanden in dieser Periode die Aushandlungen sowie die historischen Verschiebungen in Bezug auf Sicherheitswahrnehmung und soziale Selbstdisziplinierung statt.

## Schlaglichter auf die Beleuchtungsgeschichte

Der vormoderne Staat versuchte die Straßen seiner Städte nachts zu „erleuchten“. Das Verbot des Außer-Haus-Gehens nach Einbruch der Dunkelheit wurde dabei so modifiziert, dass Bielefelder Bürger*innen, die dennoch in der Dunkelheit unterwegs sein mussten, eine Laterne mit sich zu führen hatten (Büschenfeld 2000: 18). Dies hatte keinesfalls nur etwas mit persönlicher Sicherheit zu tun. Das Licht diente als Erkennungsmerkmal für Ordnung; die Einwohner*innen Bielefelds machten sich sicht- und erkennbar und „verschwanden“ nicht in der Nacht. Auch in anderen Städten wurde vonseiten der Polizeibehörden Wert auf das Mitführen einer Lichtquelle gelegt. Der Historiker Wolfgang Schivelbusch hat gerade an diesem Beispiel zeigen können, dass persönliche Sicherheit allenfalls ein Nebenaspekt war, während die polizeiliche Komponente des „Sichtbarseins“ deutlich mehr Gewicht hatte (Schivelbusch 1983: 84).

Diese Lösung des Problems der Dunkelheit erschien dem sich immer weiter konstituierenden Staat auf Dauer als nicht ausreichend. Werden die Überlegungen Foucaults zur Gouvernementalität herangezogen, so lässt sich argumentieren, dass die Beherrschung der Nacht subtiler, gefühlt „weicher“ sichergestellt werden musste. Menschen ohne Licht bei Nacht mussten bestraft, Fackel- und Laternenträger*innen überwacht, Bürger*innen an ihre Pflicht zur Sichtbarmachung erinnert werden. Darüber hinaus gab es ab dem 18. und umso mehr im 19. Jahrhundert den Wunsch nach einer vom Menschen unabhängigen und stetig funktionierenden Beleuchtung. Schon der Aufklärer Louis-Sébastien Mercier hält 1781 in seiner Beschreibung der Stadt Paris in Zusammenhang mit seiner Utopie der französischen Hauptstadt im Jahr 2240 fest: „Alles ist sehr viel sicherer geworden, seit man auf den Gedanken kam, in allen Stadtvierteln Leuchten einzuführen, die man schon von weitem sieht und die einen durch die Finsternis leiten“ (Mercier 1990 [1781]: 137).

Auch in Deutschland war die Idee einer Ausweitung der Herrschaft auf die Nacht durch Straßenbeleuchtung populär. Paul Jacob Marperger (1656–1730), ein Nationalökonom und Kameralist des 17. und 18. Jahrhunderts, schrieb im Jahr 1722 über die Notwendigkeit einer Straßenbeleuchtung zum Zweck der Sicherheit. Laternen würden überall dort gebraucht, wo in den „[…] Nacht-Zeiten viel gehens, fahrens und reitens ist, es werden aber als dann solche Laternen, sonderlich in großen Städten, in allen Straßen, auch in engen Gassen, als in welchen gemeiniglich den Nacht-Zeiten der größte Unfug vorgeht […] gesetzt“ (Marperger 1722: 8–9).

Ein Artikel aus der *Kölnischen Zeitung*, die anlässlich der Eröffnung eines Gaswerks in Paris (1819) einen kritischen Artikel veröffentlichte, gibt Einblick darüber, dass es bereits im 19. Jahrhundert fundamentale Kritik an dieser Politik gab:

„Jede Straßenbeleuchtung ist verwerflich: […] aus theologischen Gründe*n*; weil sie als Eingriff in die Ordnung Gottes erscheint. Nach dieser ist die Nacht zur Finsternis eingesetzt[…]. Dagegen dürfen wir uns nicht auflehnen, den Weltplan nicht hofmeistern, die Nacht nicht in Tag verkehren wollen […] aus philosophisch-moralischen Gründe*n*; die Sittlichkeit wird durch Gassenbeleuchtung verschlimmert. Die künstliche Helle verscheucht in den Gemüthern das Grauen vor der Finsternis, das die Schwachen von mancher Sünde abhält […] aus polizeilichen Gründen; Sie macht die Pferde scheu und die Diebe kühn […].“^3^

Neben solchen Überlegungen gab es auch marginalisierte Gruppen, die die Nacht als Rückzugsraum sahen (Beaumont 2016: 1–3). Sowohl die Nacht als auch die Beleuchtung wurden somit als ambivalent wahrgenommen und es gab keinesfalls einen Automatismus in Bezug auf die Verankerung der Gasbeleuchtung im öffentlichen Raum.

Mit dem 19. Jahrhundert wandelte sich die Beleuchtung in vielfacher Hinsicht: Als Technologie (Wandel von der Tran- zur Öl- und Gas- und schließlich zur elektrischen Lampe), als Infrastruktur wurde sie Teil der Expansion urbaner Reformprogramme sowie zu einem Element der Nacht als kommerzialisiertem Vergnügungsraum (Reulecke 1990: 172–180; Blaß 2019: 137–138; Becker 2011: 148). Unter diesen Vorzeichen verbanden sich auch Erwartungen und Ansprüche im Bereich der Sicherheit mit der Straßenbeleuchtung. So schreibt Prof. Dr. Friedrich Knapp in seiner Geschichte der Gasbeleuchtung aus den 1860er Jahren – einem Zeitpunkt, zu dem im deutschen Raum die erste Welle des Straßenbeleuchtungsbaus bereits abgeschlossen war – von den enormen Erwartungen, die an die neue Technologie gestellt wurden:

„Die öffentliche Beleuchtung der Städte ist nicht aus dem Bestreben nach Eleganz und Comfort, sondern aus der bittersten Noth der Unsicherheit der Straßen und Gassen, aus rein polizeilicher Veranlassung hervorgegangen. Diese Unsicherheit hatte besonders an grossen Orten und Residenzen mit deren wachsendem Umfang eine Höhe erreicht, welche eine Abhilfe schlechterdings nothwendig machte“ (Knapp 1866: 7).

Im letzten Drittel des 19. Jahrhundert wurde die Straßenbeleuchtung als zivilisatorische Errungenschaft und ganz selbstverständliche Lösung für das Problem nächtlicher Unsicherheit gefeiert – wie in *Die Chemie des täglichen Lebens* (1878):

„Der Tag baut – die Nacht zerstört. […] Seit Einführung einer guten Straßenbeleuchtung hat sich die öffentliche Sicherheit in gleicher Weise gehoben, wie die Zahl der Laternen sich vermehrt hat.“ (Von Hamm et al. 1878: 289–290)

Wie Rieker und Zimmermann dabei festhalten, war die moderne Gasbeleuchtung als Technologie auch deshalb von neuer Qualität, da sie über ein zentrales Netz reguliert wurde: Entindividualisiert und mit höherer Leuchtkraft war sie ein viel stärkeres Kontrollinstrument als ältere Lampen (Rieker & Zimmermann 1997: 49).

Generell entfernte die moderne Gastechnik sich aus dem Zugriffsvermögen der Bevölkerung: Kohle wurde in weit entfernten Gaswerken erhitzt und entgast, um brennbares Gas freisetzen zu können. Dieses wurde dann durch unterirdisch verlaufende Röhren an öffentliche, später auch private Anschlüsse verteilt. Die Gaslaterne war deutlich heller als ihre Vorgänger, die mit Öl funktionierten; sie war schwerer zu sabotieren, deutlich teurer in der Anschaffung und setzte ein erhebliches Maß an Infrastruktur (Gaswerk, Gasometer, Gasleitungen, eigentliche Laternen) voraus, wodurch das Unterfangen, ein gasbasiertes Beleuchtungssystem zu etablieren, teuer und äußerst zeitintensiv war (Beaugrand 1989: 6–7).

## Bielefelder Nächte: Die Dunkelheit als ordnungs- und sicherheitspolitisches Problem und die „Lösung Laterne“

Im Jahr 1832 ereignete sich in der ostwestfälischen Stadt Herford ein gewaltiger Brand, der großen Schaden anrichtete. Der Vorfall fand auch in Bielefelder Akten Eingang, da ihn die Stadtverwaltung mit einem Risiko in Verbindung brachte, das auch dort ernst genommen wurde: Der Nachtwächter hatte den Ausbruch des Feuers scheinbar nicht bemerkt.^4^ Dies wurde nicht nur von der Bielefelder Stadtverwaltung mit Sorge aufgenommen. Auch die Bürger*innen Bielefelds verlangten nach mehr Sicherheit. In den städtischen Akten wird vermerkt, es bräuchte mehr Nachtwächter, die auch mit den Turmwächtern besser kommunizieren sollten. Zeiten für die Rundgänge der Nachtwächter müssten ferner verlängert werden. Die Stadt reagierte in diesem Fall also nicht mit einer technologischen, sondern mit einer verwaltungstechnischen Lösung und ermahnte die eigenen Nachtwächter, aufmerksamer zu sein. Zum Ärger der Bürger*innen sah sie sich außerstande, mehr Nachtwächter anzustellen, nachdem eine Liste mit Kandidaten aufgestellt worden war.^5^

Der Brand von 1832 war hier ein Anlass, der das Problem der Nachtwache wieder zutage treten ließ, doch hatte es schon früher Bedenken gegeben, ob die Bielefelder Nachtwächter für die Sicherheit ausreichten: Schon im Herbst 1811 beklagten sich die Bürger*innen der Neustadt, dass es durch den gesundheitsbedingten Ausfall eines Nachtwächters nicht mehr möglich war, diesen Teil der Stadt sicher zu kontrollieren. Vor der Einführung der flächendeckenden Gasbeleuchtung in den 1850er Jahren war die Organisation der Nachtwache ein kontinuierlicher Prozess: Wiederholt wurden die Nachtwächter ermahnt oder ihre Wachzeiten verändert,^6^ zumeist auf Anregung oder Verlangen der Bielefelder Bürger*innen. Diese artikulierten ihre Ängste und das Gefühl mangelnder Sicherheit ausführlich. Im Gegensatz zur Zeit der Industrialisierung standen hier allerdings noch nächtliche Verbrechen wie Raubüberfälle und vor allem Feuergefahr im Vordergrund.^7^ Dass die städtische Obrigkeit die Nacht insgesamt als Gefahrenraum wahrnahm, zeigt sich auch darin, dass „[b]egangene Verbrechen und nächtlicher Unfug“ in gesonderten Akten geführt wurden und diesen in der Bürokratie eine eigene Qualität zugesprochen wurde.^8^

Dabei war die erste Initiative zum Aufstellen von Laternen in Bielefeld schon vor dem 19. Jahrhundert angedacht worden. Im 18. Jahrhundert kam sie allerdings nicht aus der Stadt selbst, sondern von außen: 1719 sollte auf eine Order von König Friedrich Wilhelm I. hin eine Straßenbeleuchtung mit 150 Öllaternen aufgestellt werden, wobei sich kein Hinweis findet, dass dieser Befehl tatsächlich ausgeführt wurde (Büschenfeld 2000: 18). Die Öllaternen hätten die ganze Nacht brennen sollen – länger als die spätere Gasbeleuchtung, die aus Kostengründen tatsächlich in Nächten mit hellem Mondschein nie durchweg brannte. Es lassen sich erst um 1818 lediglich drei Öllaternen in Betrieb nachweisen (Vogelsang 1988: 156–160).

In der Region Ostwestfalen gab es im frühen 19. Jahrhundert noch erhebliche Vorbehalte gegen die Straßenbeleuchtung, vor allem aus finanziellen Gründen. In Herford, einer Nachbarstadt Bielefelds, entfernte die Bürgerschaft sogar die von den Franzosen angebrachten Laternen nahezu gänzlich, da sie als Symbol der Fremdherrschaft wahrgenommen wurden und zudem befürchtet wurde, die Öl- und Tranlampen würden durch ihre schwache Helligkeit Verbrecher*innen erst animieren (Beaugrand 1989: 8).

Dies verweist auf die Vielschichtigkeit des Infrastruktur- und Sicherheitsprojekts Straßenbeleuchtung: Neben der Notwendigkeit eines wirtschaftlich profitablen Betriebs brauchte es das Engagement oder wenigstens die Zustimmung der organisierten Bürgerschaft, die die Etablierung der Beleuchtung als ihr eigenes Projekt stützte statt es als obrigkeitsstaatliches Instrument von außen nicht umzusetzen (wie in Bielefeld) oder gar rückgängig zu machen (wie in Herford). Ohne die Internalisierung des sozialdisziplinierenden Versicherheitlichungsanspruchs durch die Bürgerschaft blieb die normative Verordnung de facto wirkungslos.

Bedeutung und Ausgestaltung der öffentlichen Straßenbeleuchtung wurde daher beeinflusst von drei teils korrelierenden Aspekten: technischen Entwicklungen, ökonomischen Möglichkeiten und gewandelten Sicherheitsbedürfnissen. Diese drei Aspekte waren keineswegs statisch. Während sich die Technik beständig weiterentwickelte und ökonomische Faktoren sich veränderten, wuchs mit der Industrialisierung und Urbanisierung ein neues Sicherheitsproblem in der Dunkelheit Ostwestfalens – vor allem für die bürgerlichen Schichten.

Mitte des 19. Jahrhunderts vollzog sich in Bielefeld eine rapide Industrialisierung, die auch das soziale Gefüge massiv veränderte: Während Alt- und Neustadt von traditionellem Handwerk, aber auch weiterhin von der kaufmännischen Bürgerschaft geprägt blieben, entstand im Norden und Südosten in Jöllenbeck, Schildische und Heepen eine dynamische Textilindustrie; im Südwesten, in Gadderbaum und Brackwede, wiederum Maschinenbau und Metallverarbeitung. Gleichzeitig zog die Stadt mehr und mehr Arbeiter*innen an, die allerdings aus dem Umland nach Bielefeld pendelten. Dieser Umstand und die urbane Struktur ließen Arbeiter*innen größtenteils als ortsfremd erscheinen und verstärkten das in der Bürgerschaft ohnehin schon ausgeprägte Misstrauen und Unsicherheitsgefühl gegenüber diesen städtischen Unterschichten (Yi 1990: 18–19). Der vorherrschende Eindruck verstärkte sich noch, als Ostwestfalen im Allgemeinen und Bielefeld im Besonderen zu sozialdemokratischen Hochburgen wurden. In der Stadt entwickelte sich in den 1840ern eine rege sozialdemokratische Publikationstätigkeit. Ab 1845 kam es zu mehreren Arbeitsaufständen und Unruhen – vor allem in Schildische und entlang der Bahnlinie –, bei denen sogenannte „Ortsfremde“ eine tragende Rolle spielten (Elkar 1995: 154–178).

Die Industrialisierung Bielefelds und die daraus resultierende Bevölkerungsstruktur ließen einen zunehmenden Wunsch nach öffentlicher Sicherheit sowie die scheinbare Notwendigkeit einer Etablierung eines neuen Zeitregimes entstehen. Die westfälische Arbeiterschaft entstammte dem agrarischen oder handwerklichen Milieu und war an Arbeitsrhythmen gewöhnt, die in der Industrialisierung neu justiert wurden (Reulecke 1995: 79–105). Der Takt der Maschine und die auch in der Dunkelheit stattfindende Schichtarbeit wurden maßgebend. Die künstliche Beleuchtung – vor allem mit Gas statt Petroleum – wurde als geeignet angesehen, die Arbeitsdisziplin zu heben und gleichzeitig die Belegschaft zu einem neuen Arbeitsrhythmus zu „erziehen“ (De Koster & Reinke 2023: 48–79, 128).

Die künstliche Gasbeleuchtung stieß dabei auf einen Wunsch des Stadtbürgertums, zu disziplinieren – und zwar im ökonomischen wie im sicherheitspolitischen Sinne. Die Chance zur Umsetzung bot sich dem organisierten Stadtbürgertum durch die administrative Struktur der Stadtregierung: Mit der westfälischen Städteordnung von 1856 war dem Bürgertum die Möglichkeit zur Mitwirkung eingeräumt worden; die Magistratsverfassung teilte die Verantwortlichkeit auf. Die Stadtverordnetenversammlung stärkte das wirtschaftlich erfolgreiche und damit nach dem Wahlrecht privilegierte Stadtbürgertum. Die lokale Verwaltung war damit nicht nur zum Teil mit Menschen besetzt, die aus der Bielefelder Bürgerschaft kamen, sondern über die Stadtverordnetenversammlung konnte sie gezielt in die Schaffung neuer Infrastruktur eingreifen. Dies unterschied Bielefeld und Ostwestfalen von den preußischen Ostprovinzen mit ihren traditionellen Verwaltungsapparaten sowie vom Rheinland, wo vor allem inoffiziell die französisch geprägte Mairie-Verfassung mit einem stärkeren Bürgermeister wirksam blieb (Reulecke 1995: 83–86).

Damit waren auch die politisch-administrativen Grundlagen geschaffen, die der Bielefelder Bürgerschaft erlaubten, ihre durch Industrialisierung und demografische Verschiebung genährten Unsicherheitswahrnehmungen mittels technologisch-polizeilicher Infrastruktur zu begegnen (Ditt 2014: 247–260). Die Überforderung des sozialen Sicherungssystem und die zunehmende Angst der Mittel- und Oberschichten vor aufrührerischem und Gefährdungspotenzial verband sich mit tradierten Sorgen um die Nacht als unbeherrschtem und unbeherrschbarem Raum (Bouman 1991: 64–65; Büschenfeld 2000: 15–20; Bratvogel 1989: 11–12; Möller 2014: 463–286).

Die nächtliche Straße beherbergte jetzt nicht nur das Fremde und Unbeherrschte, sondern sie wurde konkret zum Raum der als gefährlich, aufrührerisch und unsittlich wahrgenommenen Arbeiterschaft (Schlör 1991: 53–55; Geissler 2000: 31–33).

Das Verlangen nach mehr Sicherheit und das Unsicherheitsgefühl städtischer Mittel- und Oberschichten ist dabei keinesfalls als irrational abzutun. Der Historiker Nicolas Kenny hat bereits darauf verwiesen, dass die Emotionsgeschichte der Straßenbeleuchtung und der urbanen Moderne Gefühle als historische Kräfte ernst nehmen und in der Analyse berücksichtigen sollte statt sie als unhistorische, irrationale Motive abzutun (Kenny 2017: 93).

Dass es bei der Entscheidung zur Etablierung einer weitläufigen Straßenbeleuchtung keinen Automatismus gab, zeigt auch, dass es letztlich bis 1856 dauerte, bis der Magistrat den Aufbau in Angriff nahm. Neben dem königlichen Projekt von 1818 hätte es noch andere Gelegenheiten gegeben, Gaslaternen zu installieren. So pries Baron Carl-Eugen d’Hansens von der „General-Direction der Leuchtgasbeleuchtung für Deutschland“ bereits 1840 seine Leuchtgasbeleuchtung bei der Stadtverwaltung an. Er fasste wesentliche Punkte wie folgt zusammen: „(1) […] die doppelte Lichtstärke als das beste Gaslicht (2) das nicht vorhanden sein der Gefahr einer Explosion (3) daß eine Stadt niemals in gänzliche Dunkelheit versetzt werden kann, wie solches bei der Zerstörung der Hauptleitung bei anderen Gasleitungen möglich ist, ein Umstand welcher bei Unglücksfällen und Unruhe in polizeilicher Hinsicht von größter Wichtigkeit ist […].^9^

Er brachte damit selbst die Verbindung aus neuem Licht und technischer Möglichkeit, neuer Sicherheit sowie polizeilichen Möglichkeiten zum Ausdruck, sein Vorschlag wurde jedoch weder aufgegriffen noch umgesetzt. Erst als Gasbeleuchtung ein zentrales Element innerhalb der Risikoüberlegungen Bielefelds sowie der Bielefelder Bürger*innen und ihrer Selbstregulierung wurde, wurde die Technologie Teil der Infrastrukturpolitik der Stadt (Geissler 2000: 29–45).

Die mit dem Stadtbürgertum zusammenarbeitende Verwaltung inszeniert sich dabei als Wahrerin des „Allgemeinwohls“. Im Zentrum standen Bemühungen um Hygiene, Reform des urbanen Lebens und Ausbau und Modernisierung der Stadtverwaltung (Blaß 2019: 159–160; Tarr 2019: 316–318; Schulte Beerbühl 2014: 323–330; Lenger 2009: 147–159; Heinrich & Kramer 2014: 125–133; Hofmann 1987: 31–50; Ladd 1990: 46).^10^

Dass eine Verschiebung des Sicherheitsbedürfnisses erfolgte und auch das Stadtbürgertum dem Gaslicht mehr Bedeutung zugestand, zeigt sich auch daran, dass es nach der Ablehnung des Vorschlags von 1840 bereits 1853 zwei Initiativen zur Etablierung eines Gaswerks und einer gasbetriebenen Straßenbeleuchtung gab: eine vom Magistrat und eine von 25 namhaften Bielefelder Bürger*innen. Diese hatten bereits eine beträchtliche Summe dafür aufgebracht und schlugen eine gemeinsame, privat-öffentliche Nutzung des Leuchtgases vor.^11^

Die privatwirtschaftliche Initiative und Partnerschaft war dabei ein üblicher Weg für die meisten Städte Deutschlands und des westlichen Preußens: Hohe Kosten und Aufwand machten es für die meisten Städte attraktiver, den Aufbau dieser Infrastruktur nicht selbst finanzieren zu müssen. Der Bielefelder Magistrat jedoch lehnte eine solche öffentlich-private Partnerschaft ab und vertrat stattdessen den Standpunkt, die Straßenbeleuchtung sei eine wichtige sicherheitspolizeiliche Aufgabe, die nicht an ein Privatunternehmen abgetreten werden dürfe (Matzerath 1985: 201–203; Geissler 2000: 30–31).

Er offenbarte somit, dass trotz der Absicht, auch die ebenfalls 1853 gegründete Ravensberger Spinnerei mit Gas zu versorgen, Straßenbeleuchtung eine kritische Sicherheitstechnologie war, die in die Hand der örtlichen Obrigkeit gehörte. Gleichzeitig versprach das Gaswerk auch kommunalen finanziellen Gewinn. Es zeigt sich hier, dass Sicherheitspolitik, Obrigkeitsanspruch und ökonomische Motive zusammenflossen (Geissler 2000: 31).

So trieben Bürgermeister und Magistrat die Gründung einer eigenen Gasanstalt voran – wobei sie auch die Gefahr sahen, Bielefeld könnte ins Hintertreffen geraten. Als zivilisatorischer Marker war die Gasbeleuchtung bereits so etabliert, dass auch Prestigedenken eine Rolle spielte: Die Ausbreitung der Gasbeleuchtung in Westfalen und in den Rheinprovinzen zwinge Bielefeld dazu, nachzuziehen.^12^

Trotz der privaten Initiative rechnete der Magistrat mit erheblichem Widerstand aus der Bürgerschaft gegen das Projekt. Mit Bezug auf die früheren Erfahrungen, als die Bürger*innen die Beleuchtung als ineffektiv, teuer und sogar gefährlich wahrgenommen hatten, bereitete der Magistrat zusammen mit dem zuständigen Gasingenieur Pamphlete und Argumente vor, um die Bürgerschaft zu überzeugen. Zur Verblüffung von Bürgermeister, Magistrat und Ingenieur spielten finanzielle, aber auch Sicherheitsbedenken wie die der Feuergefahr im Gegensatz zu früheren Versuchen keine Rolle mehr. Im Gegenteil – die Bürgerschaft begrüßte das Projekt in der Stadtverordnetenversammlung und in Form privater Zuschriften enthusiastisch. Hier verdeutlicht sich nicht nur ein Wandel von Sicherheitsbedürfnissen – weg von der Feuergefahr hin zur Kontrolle der Straßen und der Anwohner*innen durch Licht –, sondern auch die Tatsache, dass Bürgerschaft und städtische Obrigkeit nun ähnliche Vorstellungen des Gemeinwohls teilten.^13^

Die Bielefelder Bürger*innen arbeiteten nun gemeinsam mit dem Magistrat auf eine Straßenbeleuchtung hin, während die damit verbundene Regulierung des eigenen Verhaltens und des unmittelbaren Lebensraums akzeptiert wurde. Das Aufstandspotenzial der Unterschichten – ein klassisches Problem der Obrigkeit – ließ dabei im Stadtbürgertum die Bereitschaft entstehen, soziale Prozesse gezielt zu steuern (Gräfin zu Castell Rüdenhausen & Reulecke 1990: 61–62; Geissler 2000: 31–33). Auch in Bielefeld hatte die Reform des Städtewesens nach den napoleonischen Kriegen systematisch die Rolle des Magistrats als Interessenvertreter des Staates in den Städten gestärkt und seine polizeilichen Funktionen massiv ausgebaut (Büschenfeld 2000: 17; Birkefeld & Jung 1994: 22).

Diese Polizeifunktion ermöglichte es dem Bielefelder Magistrat, viel eher als Ordnungsmacht aufzutreten (Bratvogel 1989: 80–84). In Herford zwangen engagierte Bürger*innen ihren Magistrat regelrecht zur Einführung einer Gasbeleuchtung, nachdem sie selbst vor 50 Jahren die Laternen, die die französische Besatzungsmacht angebracht hatte, entfernt hatten: das deutlichste Zeichen für historischen Wandel in der Region in Bezug auf Verschiebung von (Un‑)Sicherheitswahrnehmungen (Beaugrand 1989: 8).

Das „Allgemeinwohl“ durch Straßenbeleuchtung zielte nicht darauf, jede*n in gleicher Weise zu inkludieren (Ithom & Eidam 2009: 16). Die Beleuchtung wurde im Interesse und auf Wunsch von Obrigkeit und Bürgertum der Alt- und Neustadt etabliert, als Mittel der Überwachung, Sichtbarmachung und Kontrolle der Arbeiterschaft und der Ortsfremden, die von diesem politischen Prozess ausgeschlossen waren. Die für den Magistrat überraschende Akzeptanz des Projekts hing eng damit zusammen, wie die Infrastruktur des Gaslichts in der Stadt verortet war. Die Belästigung durch Geruch, Rauch sowie weitere Risiken hatten die Bürger*innen der Neu- und Altstadt nicht zu fürchten: Das Gaswerk sollte auf dem sogenannten Kesselbrink, einer in den 1850er Jahren am Rand der Stadt liegenden Grünfläche, gebaut werden. In der Nachbarschaft lebten vor allem Heuerlinge, Handwerk*innen, Weber*innen und damit soziale Gruppen außerhalb der bürgerlichen Gesellschaft. Diese verfügten aufgrund des Wahlrechts und der politischen Strukturen in der Stadtverordnetenversammlung über keine Stimme und auch nicht über das soziale, kulturelle oder monetäre Kapital, um gegen den Bau vorzugehen. Als im Dezember 1853 entschieden wurde, 600 Flammen zur öffentlichen Beleuchtung anzumelden, profitierten dabei fast ausschließlich die Bewohner*innen der Innenstadt, in der die Bielefelder Bürgerschaft Wohn- und Geschäftsräume unterhielt (Geissler 2000: 32). Die Standortdebatte zeigt auch ein wesentliches Element der Selbstregulierung durch das gouvernementale Mittel der Straßenbeleuchtung. Im Allgemeinen waren Straßenbeleuchtungen nie für die Viertel vorgesehen, in denen die gefürchteten Unterschichten wohnten, obwohl diese als ein wesentlicher Grund für die Einführung in den Quellen auftauchen (Bouman 1987: 17). Die Straßenbeleuchtung wurde vielmehr in Vierteln installiert, in denen sie den Bürger*innen Sicherheit versprach, indem sie ihren Lebensraum ordnete, beleuchtete und damit reglementierte. Wird außerdem miteinbezogen, dass zentrale und deshalb beleuchtete Straßen wie die Obernstraße inzwischen auch Einkaufsstraßen waren, in denen viele Bürger*innen zwar Geschäfte hatten, aber nicht immer wohnten, kommt ein weiterer Faktor hinzu. Diese Straßen bildeten zwar bürgerlichen Lebensraum, aber eben auch eine – zumindest mögliche – imaginierte Kontaktzone zu den ebenfalls als ortsfremd angesehenen Arbeiter*innen. Die Präsenz von Geschäften verweist dabei auch auf die Bedeutung der Beleuchtung für die Kommerzialisierung der Nacht und ihren Wandel hin zum Erlebnis- und Unterhaltungsraum (Bratvogel 1989: 158; Schott 2008: 167).

Ähnlich wie Otter betont, veränderte die Präsenz der Gaslaternen auf der Obernstraße aber auch Sichtpraktiken und damit Sicherheitsbedürfnisse. Gerade die Präsenz der Beleuchtung auf der Hauptstraße ließ die Nebenstraßen nun umso risikobehafteter erscheinen. Eine Eingabe spricht dies offen an:

„Die unterzeichnenden Bewohner von der Obernstraße, bitten ganz ergebens, die Seitenstraße vom Klosterplatz von der Obernstraße aus mit Gaslaternen zu beleuchten. Die Hausbesitzer, die größtentheils Läden mit großen Spiegelscheiben haben, sind gefährdet, wenn die dunklen Nebenstraßen den finsteren und übelwollenden Menschen als Schlupfwinkel dienen und so eine Verfolgung durch Nachtwächter nicht möglich ist.“^14^

Neben den üblichen Dunkelheitsstereotypen stellt diese Eingabe eine typische Bitte um Ausweitung der Straßenbeleuchtung dar. Das Sichtbarsein ist auch hier ein Attribut von Personen, die im mehrfachen Sinn diszipliniert sind. Es ist eine Bitte um Ausbau der Regulierung, die nicht mehr nur das eigene Umfeld, sondern auch die von der Industrialisierung und Urbanisierung geschaffenen Kontaktzonen umfassen soll. Gleichzeitig offenbart die Quelle, dass Gaslaterne und Nachtwächter komplementär zusammengedacht wurden: Polizieren war immer noch auf ein Zusammenwirken von Technologie und Mensch angewiesen. In seinem klassischen Aufsatz zur preußischen Polizeigeschichte verweist Ralph Jessen bereits auf den Umstand, dass der Wandel von Nachtwache und kommunal ad-hoc organisierter Polizei zum staatlichen Organ im preußischen Westen von der kommunalen Verwaltung genutzt wurde, um eine Vielzahl von Aufgaben an sich zu ziehen. Darunter auch die Überwachung und ein Zusammengehen von Infrastruktur wie der Gasbeleuchtung und der Polizei zu einem verstärkten Disziplinierungs- und Überwachungsanspruch (Jessen 1994: 161–169).

Die Unsicherheitswahrnehmung der seit den 1850ern auf Überwachung ausgerichteten und durch neue Beleuchtungsinfrastruktur unterstützten kommunalen Polizei speiste sich nicht nur aus neuen Sehpraktiken und der Industrialisierung, sondern auch aus vermeintlich geschlechtsspezifischen Risikofaktoren:

„Die entfernten Theile des Cantons sind vielfach von Arbeitern bewohnt, die fast sämtlich die genannte Straße Abends passieren; anständigen Frauen ist es nicht möglich dann auszugehen, weil sie bei der herrschenden Dunkelheit vielfach übermüthigen Angriffen ausgesetzt sind. An Sonn- und Feiertagen Abenden herrscht auf der Wertherstraße oft wilder Lärm, begleitet von muthwilligem [sic!] Beschädigung […].“^15^

Aus den Außenbezirken kommende Arbeiter*innen werden hier als zu Kontrollierende festgeschrieben und explizit sowohl mit „Lärm“ als auch mit „muthwilligem Beschädigung“ und implizit mit sexuellen Übergriffen in Verbindung gebracht. Ihr Sichtbarmachen wird dabei als effektives Mittel zur Bekämpfung eines solchen Verhaltens präsentiert. Gleichzeitig taucht hier ein Bezug zu geschlechtsspezifischer Unsicherheit auf: „[A]nständige Frauen“ seien in der Dunkelheit in Gefahr und schutzbedürftig.

Hier zeigt sich auch eine genderspezifische Differenzierung, denn „nicht anständige Frauen“ werden klar außerhalb dieses Mechanismus gestellt. Straßenbeleuchtung kennzeichnete in der Wahrnehmung der Bielefelder Bürgern*innen also beherrschten, kontrollierten Raum mit regulierten Menschen. Ein konkretes Beispiel zur Untermauerung dieser These und der damit verbundenen Geschlechtervorstellungen findet sich in einer Eingabe zur Errichtung einer Straßenbeleuchtung am Nebeltor, die nicht nur verhindern würde, dass

„[… das] Niederstürzen von Personen an den abschüssigen Stellen, sowie in der Dunkelheit an den von der Arbeit nach Hause zurückkehrenden Näherinnen verübte[…] Rohheiten sich so wiederholen. Gegenwärtiger Antrag auf recht baldige Aufstellung einer Straßenlaterne an vorbemerkter Stelle dürfte umso mehr als gerechtfertigt erscheinen, als die Anwohner rep. Personen anderer, weniger frequentierter Straßen sich schon lange der Beleuchtung derselben zu erfreuen haben.“^16^

Die hier präsentierte Dichotomie von gefährlicher Umgebung durch Bauweise und die Gefahr durch „Rohheiten“, denen sich die anständigen Näherinnen ausgesetzt sehen, bringt den physischen und den sozialen Raum zusammen. Dabei muss die Zentralität der geschlechtsspezifischen Kodierung betont werden. Die Arbeiten von De Koster haben gezeigt, dass vor allem junge Frauen wesentlichen Anteil an der Kommerzialisierung der Nacht und dem Entstehen der nächtlichen Ökonomie hatten und deshalb verstärkt Regulierungsansprüchen ausgesetzt waren (De Koster & Reinke 2023: 45–47).

Der Wunsch nach Inklusion in den vermeintlich sicheren Raum ist auch der Wunsch, Teil der beherrschten, kontrollierten Gesellschaft mit ihren normativen Geschlechtervorstellungen und der damit verknüpften Wirtschaftsordnungen zu werden. Das Unverständnis der Anwohner*innen, dass sie als respektable Bürger*innen in der nächtlichen „Wildnis“ leben müssen, ist auch als Schritt zur Selbstregulierung zu verstehen. Menschen zwingen sich selbst in die Sichtbarkeit des Lichtkegels, um gleichfalls sicher zu sein, dass die Obrigkeit das Sichtbare sanktionieren kann und das Problem der modernen, urbanen Gesellschaft auf diese Weise löst. Die Nacht war langsam domestiziert worden und innerhalb der beleuchteten Bereiche Teil des urbanen Erlebnisraums geworden. Neue Unsicherheiten taten sich damit auf, nun, da Verkehr und Wegqualität ebenso durch Licht gesichert werden mussten.

Der Wunsch nach Sichtbarmachung und mehr Verkehrssicherheit wird nun gleichberechtigt neben der Kontrolle der Arbeiter*innen genannt: Die Wertherstraße sei viel befahren (3–4 Wagen hintereinander), das Trottoir sei ungenügend. Von respektablen Bürger*innen wurde so neben der Internalisierung der Sicherheits- und Überwachungslogiken auch ein selbstbewusstes, staatsbürgerliches Anspruchsdenken, ein Recht auf die Straßenbeleuchtung formuliert. Das Fehlen dieser würde „die öffentliche Sicherheit gefährden und die Anwohner, welche mit den übrigen Stadtbewohnern Lasten tragen, in ihren Rechten sehr beeinträchtigen.“^17^ Das Vertrauen der Bürger*innen in die Beleuchtung war dabei groß und sie hielten auch nicht mit ihren Ansprüchen zurück: „Die öffentliche Sicherheit wird sich heben und die zahlreichen Anwohner werden endlich einen praktischen Erfolg ihrer Steuerquote sehen.^18^

In ihrer Antwort reagierten Oberbürgermeister und Magistrat eher gereizt auf die Eingabe, wobei sie nicht die Wirksamkeit des Lichts, sondern das ihr zugrunde liegende Anspruchsdenken kritisierten und betonten, aus Steuerzahlungen würde sich keinerlei Anrecht auf Beleuchtung ableiten.^19^ Teilweise nahmen Bürger*innen deshalb in Eigeninitiative die Ausweitung der Beleuchtung vor. Am Kesselbrink stellten Anwohner*innen drei Laternen auf, um „mehrfach vorgekommene Unsittlichkeiten an der unbeleuchteten Promenade abzuhalten“^20^. Auch hier zeigt sich nicht nur die Vorstellung, dass Gaslicht Sittlichkeit fördere, sondern auch, dass nächtlicher Stadtraum reguliert werden müsse, um für respektable Bürger*innen zugänglich zu sein.^21^

Mit der Obrigkeit zusammenzuarbeiten, ihre Maßnahmen zu fördern und diese gleichsam als die eigenen zu verstehen, ist ein wesentliches Element in der Selbstregulierung, nachdem staatliche Dispositive anerkannt wurden. In diesem Fall sogar so weit, dass die Bürger*innen sich an den Kosten beteiligen wollten.^22^ Diese exemplarisch ausgewählten Quellen sollen verdeutlichen, nach welchem Muster die Selbstregulierung in Bielefeld ablief. Sie ging einher mit neuen technologischen Möglichkeiten bei gleichzeitigem Rückgriff auf etablierte Narrative und Wissensarchive der nächtlichen Unsicherheit. Die Beschreibung der eigenen Nachbarschaft als respektabel und des Risikos und der Unsicherheit der Dunkelheit, die „zu allem nächtlichen Unfug benützt wird“^23^, formuliert den Wunsch nach Kontrolle, in der evidentes Risiko, technologisches Wirken, Selbst- und gesellschaftliches Interesse nicht mehr zu trennen waren.

Dabei war Licht nicht gleich Licht: Gaslicht wurde eine höhere Sicherheitswirkung zugestanden als anderen Leuchtquellen. Es machte beispielsweise nicht nur einen Unterschied auf der Ebene der Lichtstärke, ob eine Stadt Öl‑, Gas- oder Kerosinlaternen nutzte. In imperialen und kolonialen Kontexten der osmanischen Provinz stand die Kerosinlaterne für mangelnde Aufmerksamkeit und Marginalisierung durch das imperiale Zentrum, die Gasbeleuchtung wiederum als Symbol für gouvernementale Intervention und als Marker für Zivilisation (Wishnitzer 2020: 165–178; Martland 2002: 224–227).

Auch in Bielefeld strukturierte unterschiedliches Licht den Stadtraum: Als die Eingabe, am Nebeltor Gaslicht anzubringen, zunächst abgelehnt wurde, schlugen die Anwohner*innen dem Magistrat stattdessen vor, doch „wenigstens“^24^ eine oder zwei Petroleumlampen anzubringen. Diese seien zwar noch nicht genügend, aber ein möglicher Kompromiss.

Auch die Industrie, die wesentlich vom Gaslicht profitierte, war anders als noch in den 1850er Jahren nicht mehr mit der Lichtqualität zufrieden. Im März 1869 schrieb die Firma Baer & Rempel an den Magistrat, dass das Gaslicht für ihre Fabrik gänzlich ungeeignet sei. Das Rohr sei zu klein, die Lichtstärke nicht ausreichend, der Preis zu hoch. Auch hier wurde kritisiert, dass Petroleumlampen genutzt werden mussten, um die Missstände zu kompensieren.^25^

Widerstand gegen das Gaswerk in der Bürgerschaft formierte sich zum ersten Mal 1879, als es erweitert wurde. 200 Bielefelder Bürger*innen protestierten, da die Erweiterung an der Herforder Straße nunmehr in einem von ihnen bewohnten Gebiet liegen sollte. Die Belästigung durch Geruch, Rauch und Ruß, die vorher die Anwohner*innen des Kesselbrinks und damit nicht-bürgerliche Schichten betroffen hatte, würde nun also auch die Bürgerschaft betreffen. Die Umweltprobleme im innerstädtischen Raum, die durch das Gaswerk verursacht wurden, gerieten damit auf die Agenda der Stadtverordnetenversammlung, die als Interessenvertretung der kaufmännisch geprägten Bürgerschaft in der Tat eine Verlegung des Gaswerks an den Stadtrand erreichte – allerdings erst 1890 und damit über zehn Jahre nach der Protestnote der Anwohner*innen der Herforder Straße. Diese hatten vor allem gesundheitliche und hygienische Gründe angeführt, da die „Ausdünstungen“ enorm „schädlich“ seien (Geissler 2000: 38–40).

Es zeigt sich, dass dem Gaslicht eine enorme sicherheitspolitische Bedeutung beigemessen und die Ausweitung der Beleuchtung sowie ihr kontrollierendes, raum- und verhaltendisziplinierendes Potenzial immer weniger infrage gestellt wurde, aber gleichzeitig Ansprüche an das Licht stiegen, Teilhabe an der Beleuchtung zum Ausweis bürgerlicher Zugehörigkeit wurde und Umweltproblematiken auf die städtischen Unterschichten der Handwerker*innen, Heuerlinge und Weber*innen abgewälzt werden sollten.

Die aufgezeigten Kontinuitäten und Brüche in Bezug auf Zuschreibungen der kaufmännischen, innerstädtischen Bürgerschaft auf die Straßenbeleuchtung und ihren sicherheitspolitischen Charakter hingen neben der Urbanisierung auch damit zusammen, dass die Bielefelder Innenstadt – wie anderswo auch – mehr und mehr selbst nachts zu einem Erlebnis- und Konsumraum wurde, der gerade deshalb durch Gaslicht und eben nicht durch Petroleum sichtbar und gleichsam legitim gemacht werden musste. Umwelt- und Hygieneprobleme traten erst in den Vordergrund der Debatten und Auseinandersetzungen in den städtischen Gremien, als diese die Bürgerschaft und nicht mehr nur die ständischen Unterschichten betrafen.

Interessant sind auch die Leerstellen dieses Prozesses zwischen 1853 bis in die 1890er Jahre: Die Brand- und Explosionsgefahr, die beispielsweise durch Privatanschlüsse und das Nutzen von Leuchtgas bestand, wurde erst in den 1890ern artikuliert und auch dann in Lehrbroschüren des Gaswerks über den richtigen Umgang mit dem heimischen Anschluss beschrieben (Geissler 2000: 36, 40).

Die Zusammenarbeit des Magistrats als Schnittstelle von Stadtregierung, Polizeiaufgaben und sich zunehmend professionalisierender Verwaltung und Stadtverordnetenversammlung als politische Vertretung des Stadtbürgertums und wesentliches Element der Etablierung der Bielefelder Straßenbeleuchtung endete mit der Jahrhundertwende. Der Magistrat begann um 1900 mit einer einheitlichen, auf stadtplanerischen Gesamtkonzepten beruhenden Infrastrukturpolitik, die ein Mitwirken der Stadtverordneten und damit der Bürgerschaft nicht mehr im gleichen Maße vorsah. Mit dem Wechsel zum 20. Jahrhundert hatte der Magistrat nicht nur ein technokratisches Selbstverständnis, sondern ein Fachwissen ausgebildet, das der Institution neues Selbstbewusstsein gab, die Infrastruktur unabhängig von den Stadtverordneten zu planen. Stadtgestaltung sollte in Bielefeld in Zukunft in die Hände von Experten*innen gelegt werden, die durch Studium und direkte Verantwortlichkeit dem Bürgermeister gegenüber in die Lage versetzt werden sollten, eine bisweilen „rücksichtslose“ (Möller 2014: 477) Modernisierungspolitik zu fahren.

## Fazit: Bielefelder Straßenbeleuchtung als Bestandteil gouvernementaler Politik

Die Nacht wurde im 19. Jahrhundert als Risikoraum begriffen und gerade die urbane Bevölkerung des Stadtbürgertums verlangte nach mehr Sicherheit durch die Etablierung einer flächendeckenden Gasbeleuchtung. Die Laterne war eine Sicherheitstechnologie und zugleich ein Disziplinarobjekt, das zur Selbstregulierung führte. Ihre Geschichte ist keine Geschichte einer alternativlosen „Selbstverständlichkeit“, sondern eines obrigkeitsstaatlichen und bürgerlichen Herrschaftsinstruments, das am besten in seiner historischen Entwicklung gefasst werden kann, wenn sie im Rahmen der Disziplinargesellschaft und der Gouvernementalität im Sinne Foucaults betrachtet wird. Sie erfüllte dabei den Zweck mehrfacher Versicherheitlichung – erstens als Sichtbarmachung von als gefährlich, aufrührerisch und undiszipliniert wahrgenommenen sozialen Gruppen wie Arbeiter*innen, Frauen der nächtlichen Ökonomie und damit außerhalb einer imaginierten „Anständigkeit“, sowie von Ortsfremden. Zweitens als Versicherheitlichung der Nacht als Erlebnisraum, in dem Verkehrs- und körperliche Sicherheit gewährleistet wurde sowie Geschäfte und kommerzielle Unternehmungen gesichert werden konnten. Drittens als die ökonomische Versicherheitlichung durch die Neujustierung und Umerziehung der handwerklich-ländlich geprägten Arbeiterschaft zu neuen Rhythmen der Arbeit. Der Wunsch nach Licht und Sichtbarkeit, der bis in die späten 1840er und frühen 1850er Jahre nur schwach ausgeprägt war, nahm in dem Maße zu, wie mit dem Voranschreiten der Urbanisierung als fremd empfundene Arbeiter*innen in die urbanen Räume vordrangen, in denen die Bürgerschaft lebte und arbeitete. Der frühere Widerstand gegen die Sichtbarmachung wurde zugunsten neuerer Sicherheitsbedürfnisse aufgegeben und Logiken der Selbst- und Fremdkontrolle wurden internalisiert.

Die Dunkelheit war seit der Frühen Neuzeit ein ordnungs- und sicherheitspolitisches Problem für Obrigkeiten sowie eine Gefahr für weite Teile der Bevölkerung. Dabei war die Nacht mit ihrer Dunkelheit ein segmentierter Raum: Rückzugsort für marginalisierte Gruppen oder Standort einer teils unsanktionierten Ökonomie. Licht wurde mit Ordnung und Rechtschaffenheit assoziiert; Sichtbarsein war ein Attribut respektabler sozialer Schichten, die die Dunkelheit der nächtlichen Straße misstrauisch beäugten. Schon früh wurde dabei künstliches Licht als Lösung diskutiert. Der Obrigkeitsstaat des Absolutismus zwang seine Untertan*innen in Bielefeld und anderswo zum Sichtbarsein. Laternenpflicht und Öllampen waren dabei noch keine Sicherheitstechnologien für die/den Einzelne*n, sondern Indikatoren, an denen sich zeigte, wie die Obrigkeit Kontrolle ausübte (Kenny 2017: 97). Individuelle Laternen und Öllampen gaben ihre Träger*innen als gehorsame und gleichsam sichere Elemente auf der nächtlichen Straße aus.

Der moderne Staat inkludierte neue Technologien wie die Gasbeleuchtung in diesen Anspruch, der weniger individuell als vielmehr auf ganze Bevölkerungsgruppen ausgeweitet verstanden wurde. Gemäß neu formulierter Ansprüche an Polizierungspraktiken und an die Lösung sozioökonomischer wie politischer Problemstellungen durch Infrastruktur vollzog sich eine Versicherheitlichung von Raum und Bevölkerung durch das Sichtbarmachen und Sichtbarwerden von beiden gleichermaßen.

Auch in Bielefeld orientierten sich die Bedürfnisse der sogenannten städtischen Allgemeinheit „nicht an der Allgemeinheit der städtischen Einwohner, sondern sie bleiben auf die besitzende und gewerbetreibende Bürgerschaft zugeschnitten“ (Büschenfeld 2000: 15). Ergänzt werden muss hier die auch geschlechtsspezifische Dynamik, die Bielefelderinnen als entweder gefährdete anständige Frauen und damit schutzbedürftig präsentierte oder als sichtbar – und damit überwachbar – zu machende Unruhestifter*innen. Der Staat in Form des Magistrats als auch die seit Mitte des 19. Jahrhunderts als dezidiert staatliches Organ auftretende Polizei verstärkten ihren Zugriff auf die Bürgerschaft, die ihrerseits die Etablierung der Sicherheitstechnologie Gaslaterne vorantrieb. Die Bürger*innen regulierten sich selbst, baten um den Ausbau der seit Jahrhunderten mit Herrschaft assoziierten Laterne und wünschten deren Licht in ihrer nächsten Umgebung als auch in der Nähe ihrer Unternehmen. Hier zeigt sich in Bielefeld wie andernorts eine Verschiebung von Sehpraktiken: Petroleum wurde als defizitär wahrgenommen – sowohl als der Sicherheit dienende Lichtquelle wie auch zur Beleuchtung der Fabrikhallen. Die Ausweitung der Straßenbeleuchtung sorgte ferner dafür, dass nicht beleuchtete Straßenzüge als unsicher wahrgenommen wurden, was die Stadt neu strukturierte.

Die Geschichte der Gasbeleuchtung stellt deshalb ein Beispiel von Infrastrukturgeschichte dar, die untermauert, dass disziplinierende Herrschaftsinstrumente keinesfalls im Sinne Grabers nur im Top-down-Verfahren etabliert wurden, sondern dass im Sinne Foucaults die graduelle Akzeptanz und Übernahme obrigkeitsstaatlicher Projekte durch breite Teile der staatstragenden Bevölkerungsgruppen zentral war.

Die Erweiterung der Geschichte der Straßenbeleuchtung um Lokal‑, Meso- und Mikroperspektiven abseits der Metropolen eröffnet einen Zugang zu einer innovativen historischen Sicherheits- und Technikforschung, die das Potenzial hat, in eine Vielzahl von Debatten zu intervenieren.

## Danksagung

Ich danke Carolyn Taratko und der NTM-Redaktion für ihre Unterstützung, das Lektorat und ihr Entgegenkommen während der Publikationsphase sowie Jürgen Büschenfeld für die Hilfe bei der ursprünglichen Quellenrecherche und Lena Gumpert für Hilfe bei der Transkription der Archivquellen. Ferner danke ich Christine Franke, Margret Haas und zwei anonymen Gutachter*innen für ihre Korrekturen und Anmerkungen zu früheren Versionen dieses Aufsatzes.

## Footnotes

1 https://www.rbb24.de/panorama/beitrag/2022/08/interview-dunkelheit-angst-weniger-licht-peter-walschburger.html (28.03.2023).

2 Siehe hierzu die Rezensionen von Arnd Bauerkämper (2019): 438–439.

3 Zit. n. Rebske (1962,  37).

4 StAB, 100,002/Ältere Akten, 179 (Die Anstellung der Nachtwächter 1811–1880), Blatt 21.

5 StAB, 100,002/Ältere Akten, 179 (Die Anstellung der Nachtwächter 1811–1880), Blatt 24.

6 StAB, 100,002/Ältere Akten, 179 (Die Anstellung der Nachtwächter 1811–1880), Blatt 17–20.

7 StAB, 100,002/Ältere Akten, 179 (Die Anstellung der Nachtwächter 1811–1880), Blatt 16.

8 StAB, 130,004/Ältere Akten, 2669 (Amt Heepen, Begangene Verbrechen und nächtlicher Unfug).

9 StAB, 100,002/Ältere Akten, 1664 I (Gasbeleuchtung), Blatt 2.

10 Als grundlegende Studie kann hier auf Hofmann (1987) verwiesen werden.

11 StAB, 100,002/Ältere Akten, 1664, 12.11.1853, Aktiengesellschaft an Magistrat.

12 StAB, 100,002/Ältere Akten, 1664 II (Gasbeleuchtung), 326.

13 StAB, 100,002/Ältere Akten, 1664, Prof. Kloeden: „Gutachten über den Grad der Feuergefährlichkeit einer Gasbeleuchtungsanstalt“; StAB, 100,002/Ältere Akten, 1664, Prof. Schubarth: B; StAB, 100,002/Ältere Akten, 1664, Zuschrift 06.12.1853.

14 StAB, 100,002/Ältere Akten, 1664 I (Gasbeleuchtung), undatiert (vermutlich 1860), Blatt 186.

15 StAB, 100,002/Ältere Akten, 1664 II (Gasbeleuchtung), 1864, Blatt 502.

16 StAB, 100,002/Ältere Akten, 1664 II (Gasbeleuchtung), 1869, Blatt 507.

17 StAB, 100,002/Ältere Akten, 1664 II (Gasbeleuchtung), 1871, nicht paginiert.

18 StAB, 100,002/Ältere Akten, 1664 II (Gasbeleuchtung), 1864, Blatt 502.

19 StAB, 100,002/Ältere Akten, 1664 II (Gasbeleuchtung), 1864, 17.10.1871.

20 StAB, 100,002/Ältere Akten, 1664 II (Gasbeleuchtung), 1864, Blatt 433.

21 Öffentliche Anzeigen der Grafschaft Ravensberg, Kreisblatt des Kreises Bielefeld 1860; Öffentliche Anzeigen der Grafschaft Ravensberg Jahresberichte in der Verwaltung und der Stand der Gemeinde-Angelegenheiten der Stadt Bielefeld 1861.

22 StAB, 100,002/Ältere Akten, 1664 II (Gasbeleuchtung), 1864, Blatt 433.

23 StAB, 100,002/Ältere Akten, 1664 II (Gasbeleuchtung), 1860, Blatt 352.

24 StAB, 100,002/Ältere Akten, 1664 II (1817), nicht paginiert.

25 StAB, 100,002/Ältere Akten, 1664 II, 1869, Blatt 494.
